# Nucleotide-mediated SPDEF modulates TFF3-mediated wound healing and intestinal barrier function during the weaning process

**DOI:** 10.1038/s41598-018-23218-4

**Published:** 2018-03-19

**Authors:** Sang In Lee, In Ho Kim

**Affiliations:** Department of Animal Resource and Science, Dankook University, Cheonan, Chungnam 330–714 Republic of Korea

## Abstract

Most alterations during weaning involve physiological changes in intestinal structure and function. Here, we evaluated the molecular mechanisms regulating the effects of nucleotides on weaning. Nucleotide treatment induced Trefoil factor 3 (TFF3) expression and IPEC-J2 cell growth and reduced wound width. Treatment with nucleosides and TFF3 in lipopolysaccharide-challenged IPEC-J2 cells increased intestinal transepithelial electrical resistance and decreased intestinal permeability. Additionally, nucleosides improved intestinal barrier function through induction of TFF3-mediated phosphatidylinositol 3-kinase/Akt, extracellular signal-regulated kinase 1/2, p38, and Janus kinase/signal transducer and activator of transcription signaling pathways. Among selected differentially expressed genes, SAM pointed domain containing ETS transcription factor (SPDEF) expression was elevated by nucleotides in a concentration-dependent manner. Moreover, SPDEF directly regulated TFF3 expression via binding to the promoter. *In vivo*, nucleotide supplementation improved growth performance, serum stress levels, and intestinal morphology. Our findings provide insights into the molecular mechanisms of intestinal development during weaning in pigs.

## Introduction

The suckling to weaning transition is one of the most stressful events in pig development; pigs experience significant physiological, environmental, and social challenges that can contribute to intestinal and immune system dysfunction, resulting in reduced pig health, growth, and feed intake^1,2^. Pigs must resume eating and growing as soon as possible after the weaning transition because reduced weight gain during the weaning transition impacts the total days to market in pig production^3^. Thus, to minimize the adverse effects of weaning stress, it is critical to utilize appropriate health, nutrition, and management strategies and to improve swine productive measures all the way to market weight.

The gastrointestinal tract (GIT) plays critical roles in the digestion and absorption of nutrients; maintenance of bodily fluid balance to achieve proper viscosity of the luminal contents; secretion of digestive enzymes, mucin, and immunoglobulins; and maintenance of barrier function for the host against harmful pathogens and antigens^4,5^. One of the most dramatic alterations mediating the weaning transition is physiological changes in the structure and function of the intestine, including shortening of the villi (villous atrophy) and increased crypt depth (crypt elongation) during the weaning transition^2,6^. These physiological changes can impact the function of the small intestine, including digestion, absorption, secretion, and maintenance of barrier function, which may contribute to postweaning growth^1^. Thus, it is important to minimize physiological changes in the small intestine during the weaning transition to maximize pig production.

As the building blocks of DNA and RNA synthesis, nucleotides (NTs) are intracellular compounds that participate in numerous biochemical processes and are absorbed by the intestinal epithelium mainly in the form of nucleosides (NSs)^7,8^. Exogenous NTs have positive effects under conditions of limited *de novo* NT synthesis, such as rapid growth during the neonatal period and intestinal recovery after injury and stress^9,10^. Thus, we expect that NTs may be essential nutrients during the weaning transition because the weaning transition is the most active period for the recovery of small intestinal epithelial cells.

In this study, we aimed to evaluate the effects of exogenous NTs during the weaning transition using gene expression profiling of the small intestine after dietary treatment with NTs. Genes that were significantly regulated by NTs were examined further to investigate the regulatory functions for small intestinal development in pigs.

## Results

### Identification and validation of differentially expressed genes (DEGs)

**Figure 1:**
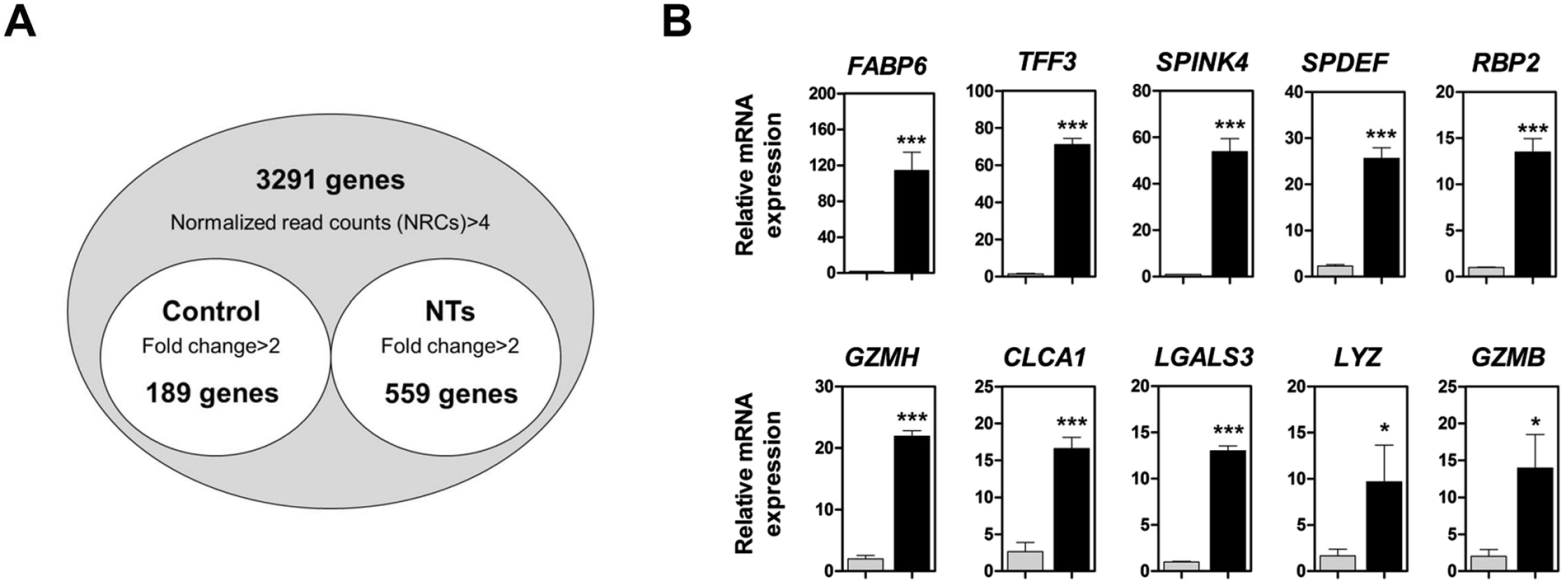
Gene expression profiling of the small intestine after dietary treatment with NTs for 14 days. (**A**) Venn diagram illustrations of genes found to be differentially expressed with or without NT treatment in the small intestine. The common genes from quanti-seq experiments were upregulated at least 2-fold (*P* < 0.05). (**B**) Quantitative expression analysis of genes that were highly expressed following NT treatment in the small intestine (n = 3). Real-time PCR analysis was conducted in triplicate and normalized to the expression levels of glyceraldehyde 3-phosphate dehydrogenase (*GAPDH*). Significant differences between control and treatment groups are indicated as ***P < 0.001, **P < 0.01, and *P < 0.05. Error bars indicate standard errors (SEs) of triplicate analyses.

To identify certain DEGs, we compared the gene expression profiles of small intestinal tissues, with or without NT treatment, for 14 days. We identified 748 annotated DEGs, among which 559 were upregulated and 189 were downregulated (Fig. 1A and Supplemental Tables 1–4). To verify the expression of DEGs, we analyzed the expression of the top 10 DEGs using quantitative reverse transcription polymerase chain reaction (qRT-PCR) in the small intestine with or without NT treatment (Fig. 1B). *FABP6* (*P* < 0.001), *TFF3* (*P* < 0.001), *SPINK4* (*P* < 0.001), *SPDEF* (*P* < 0.001), *RBP2* (*P* < 0.001), *GZMH* (*P* < 0.001), *CLCA1* (*P* < 0.001), *LGALS3* (*P* < 0.001), *LYZ* (*P* < 0.05), and *GZMB* (*P* < 0.05) were upregulated in the small intestine following NT treatment compared with that without NT treatment (Fig. 1B).

**Figure 2:**
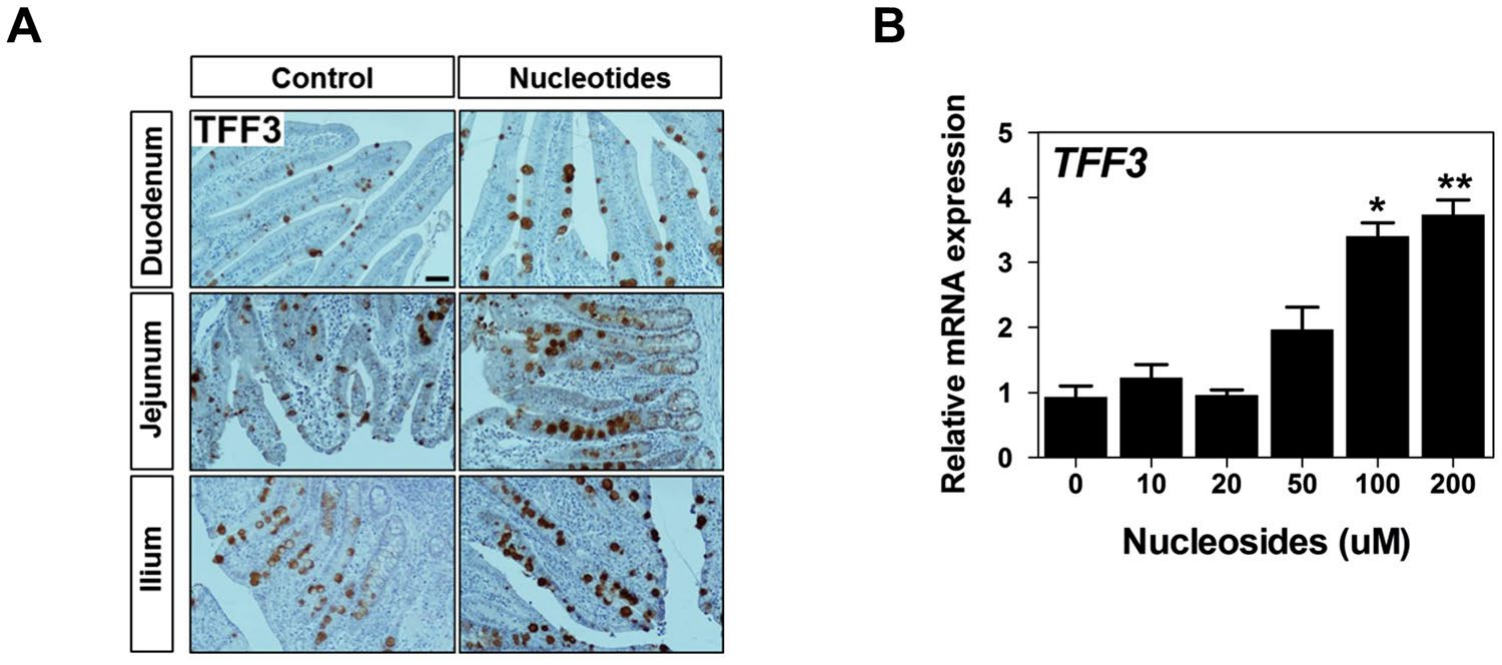
Nucleosides regulated the expression of *TFF3*. (**A**) Immunohistochemical staining of TFF3 in the duodenum, jejunum, and ileum with or without NT treatment. Scale bar: 50 μm. (**B**) IPEC-J2 cells were incubated with varying concentrations of NSs, and ectopically induced expression of TFF3 was analyzed (n = 3). Significant differences between the control (0 μM) and treatment groups are indicated as **P < 0.01. Error bars indicate standard errors (SEs) of triplicate analyses.

Next, based on the function of TFF3 in the intestine, we analyzed tissue localization of trefoil factor 3 (TFF3) in the duodenum, jejunum, and ileum with or without NT treatment (Fig. 2A). Immunoreactive TFF3 was readily detected within goblet cells in the duodenum, jejunum, and ileum with or without NT treatment along the overlying mucosal surface, reflecting secretion into the lumen. Notably, however, NT treatment ectopically induced TFF3 expression in goblet cells of the duodenum, jejunum, and ileum compared with that without NT treatment.

### NSs regulated TFF3-mediated wound healing

The effects of various concentrations of NSs on the expression of *TFF3* were investigated. After 24 h of incubation of IPEC-J2 cells with varying concentrations of NSs (10, 20, 50, 100, and 200 µM), TFF3 mRNA and protein levels were elevated in a concentration-dependent manner. At concentrations of 100 and 200 µM, NSs markedly stimulated the expression of *TFF3* in IPEC-J2 cells (*P* < 0.05; Fig. 2B). Based on these results and previous reports, we used 100 μM NSs and 100 ng/μL recombinant porcine TFF3 in further experiments.

**Figure 3:**
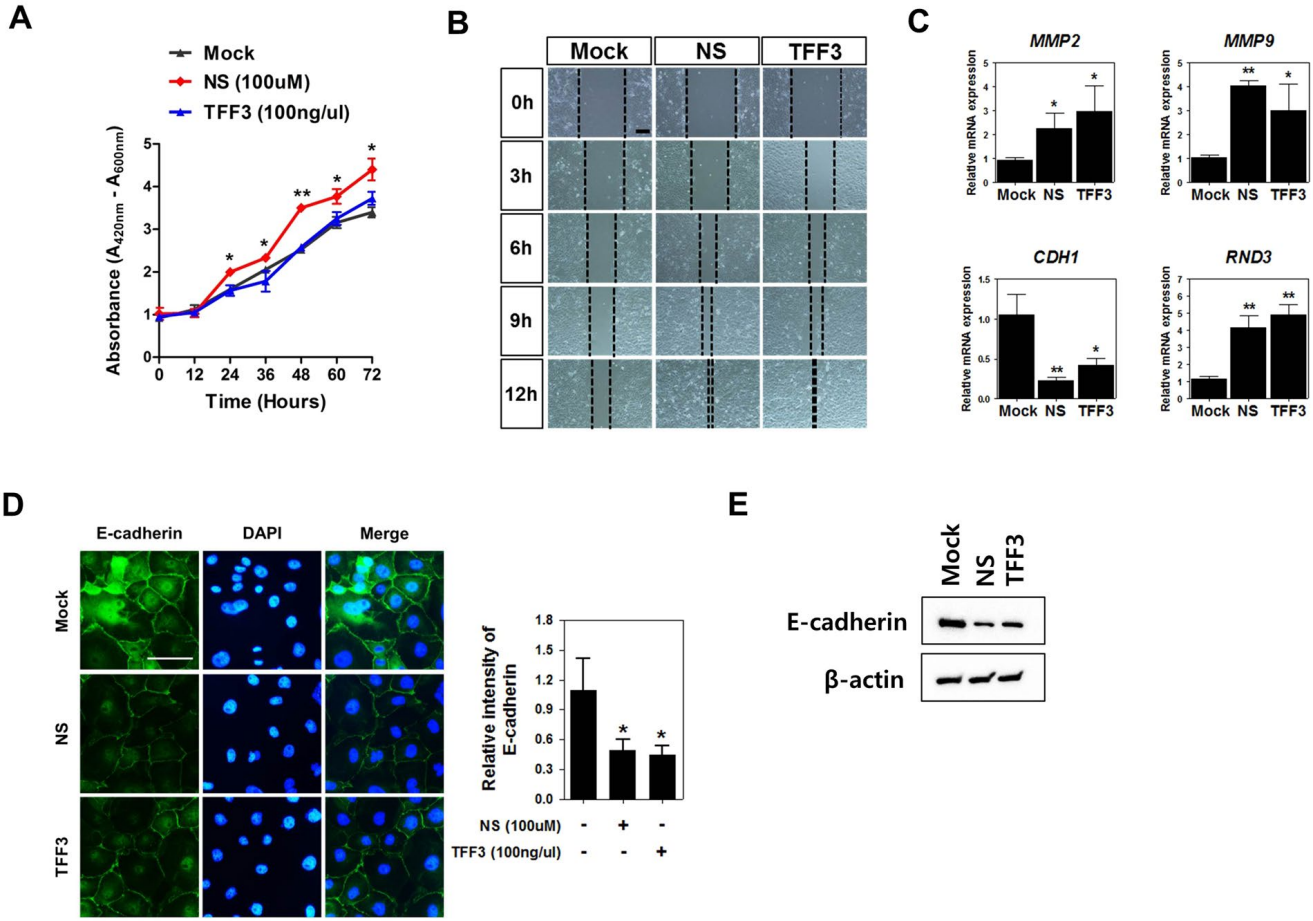
Nucleosides regulated TFF3-mediated wound healing. (**A**) The quantity of viable cells was determined at 12, 24, 36, 48, 60, and 72 h after treatment with NSs (100 μM) and TFF3 (100 ng/µL) using WST-1 assays (n = 3). Significant differences between control and treatment groups are indicated as **P < 0.01 and *P < 0.05. (**B**) Effects of NSs and TFF3 on migration in IPEC-J2 cells at 0, 3, 6, 9, and 12 h (n = 3). (**C**) Expression of wound healing-related genes, such as *MMP2*, *MMP9*, *CDH1*, and *RND3*, was analyzed with or without NSs or TFF3 in IPEC-J2 cells by real-time PCR. (**D**) Immunocytochemistry of E-cadherin in IPEC-J2 cells was modulated by NSs and TFF3 treatment. Immunofluorescence staining for E-cadherin (green) showed a membranous expression pattern. Nuclei were stained with 4′,6-diamidino-2-phenylindole (DAPI; blue). Fluorescence intensities were quantified using the threshold function of Image J software. (**E**) Western blotting assay of E-cadherin in IPEC-J2 cells was performed following NS and TFF3 treatment. Significant differences between control and treatment groups are indicated as *P < 0.05. Error bars indicate standard errors (SEs) of triplicate analyses.

To evaluate the effects of NSs and TFF3 on intestinal wound healing, which is dependent on the precise balance of migration, proliferation, and differentiation of epithelial cells adjacent to the wounded area, we investigated the proliferation and migration capacity of IPEC-J2 cells following treatment with NSs and TFF3. Addition of NSs to the medium stimulated IPEC-J2 cell growth in time‐dependent manner; however, TFF3 treatment did not affect IPEC-J2 cell growth (Fig. 3A). NSs and TFF3 significantly reduced wound width in a time-dependent manner using scratch assays in IPEC-J2 cells (Fig. 3B).

To verify the effects of NSs and TFF3 treatment on differentiation, we analyzed the expression of wound healing-related genes, e.g., matrix metalloproteinase (*MMP*) 2, *MMP9*, cadherin 1 (*CDH1*), and Rho family GTPase 3 (*RND3*), with or without NSs or TFF3 in IPEC-J2 cells (Fig. 3C). NS treatment increased the expression of *MMP2* (*P* < 0.05), *MMP9* (*P* < 0.01), and *RND3* (*P* < 0.01) and decreased the expression of *CDH1* (*P* < 0.01). Additionally, TFF3 treatment increased the mRNA expression of *MMP2* (*P* < 0.05), *MMP9* (*P* < 0.05), and *RND3* (*P* < 0.01) and decreased the expression of *CDH1* (*P* < 0.05) in IPEC-J2 cells. Immunostaining and western blotting assays demonstrated that NSs and TFF3 treatment for 6 h reduced the expression of E-cadherin, which was regularly distributed along the cell borders of IPEC-J2 cells (*P* < 0.05; Fig. 3D and E).

### Wound healing was mediated via the phosphatidylinositol 3-kinase (PI3K)/Akt, extracellular signal-regulated kinase (ERK) 1/2, p38, and Janus kinase (JAK)/signal transducer and activator of transcription (STAT) signaling pathways

**Figure 4:**
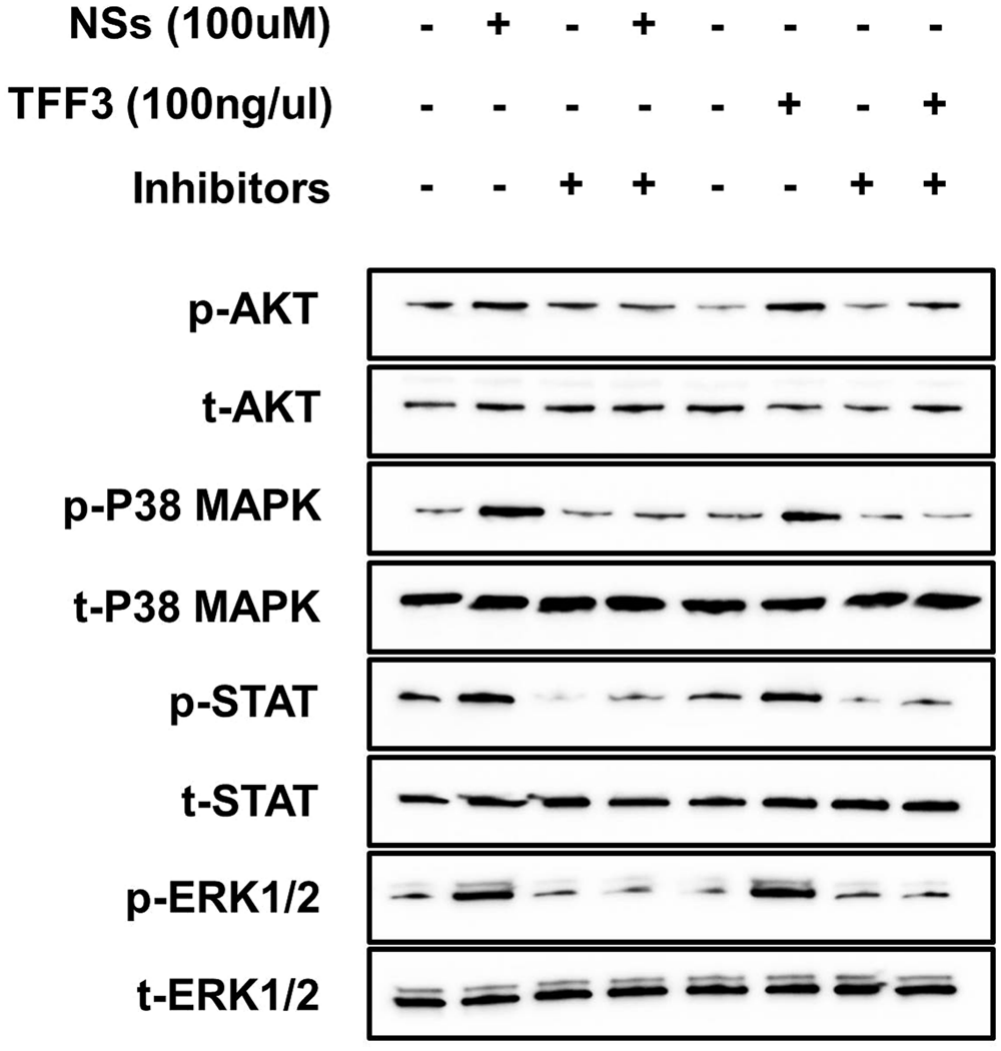
Analysis of PI3K/Akt, ERK1/2, p38, and JAK/STAT signaling pathways in IPEC-J2 cells treated with or without NSs or TFF3 (n = 3). IPEC-J2 cells were treated with 100 μM NSs and 100 ng/µL TFF3 for 2 h, and the phosphorylated forms of Akt (Ser473), p38 (Thr180/Tyr182), ERK1/2 (Thr202/Tyr204), and JAK/STAT (Ser727) were detected by western blot analysis. PI3K/Akt (LY2944002), ERK1/2 (U0126), p38 (SB202190), and JAK/STAT (STA-12) were used as inhibitors of corresponding signaling pathways. Whole images of blots are presented in the supplemental information for Fig. 4.

To verify the underlying mechanisms through which TFF3 mediated NSs during intestinal wound healing, we investigated the functional roles of PI3K/Akt, ERK1/2, p38, and JAK/STAT signaling pathways (Fig. 4). Treatment of IPEC-J2 cells with NSs and TFF3 increased Akt phosphorylation, and inhibition by LY294002 decreased Akt phosphorylation following treatment with NSs and TFF3 in IPEC-J2 cells compared with that in the untreated control. Treatment of IPEC-J2 cells with NSs and TFF3 also increased p38 phosphorylation, and inhibition by SB202190 decreased induction of p38 phosphorylation following treatment with NSs and TFF3 in IPEC-J2 cells compared with that in the untreated control. Treatment of IPEC-J2 cells with NSs and TFF3 increased STAT phosphorylation, and inhibition by STA-12 decreased induction of STAT phosphorylation following treatment with NSs and TFF3 in IPEC-J2 cells. Finally, ERK1/2 phosphorylation was induced by NSs and TFF3 and inhibited by U-0126 compared with that in control cells.

### Protective effects of NSs and TFF3 on the intestinal barrier function

**Figure 5:**
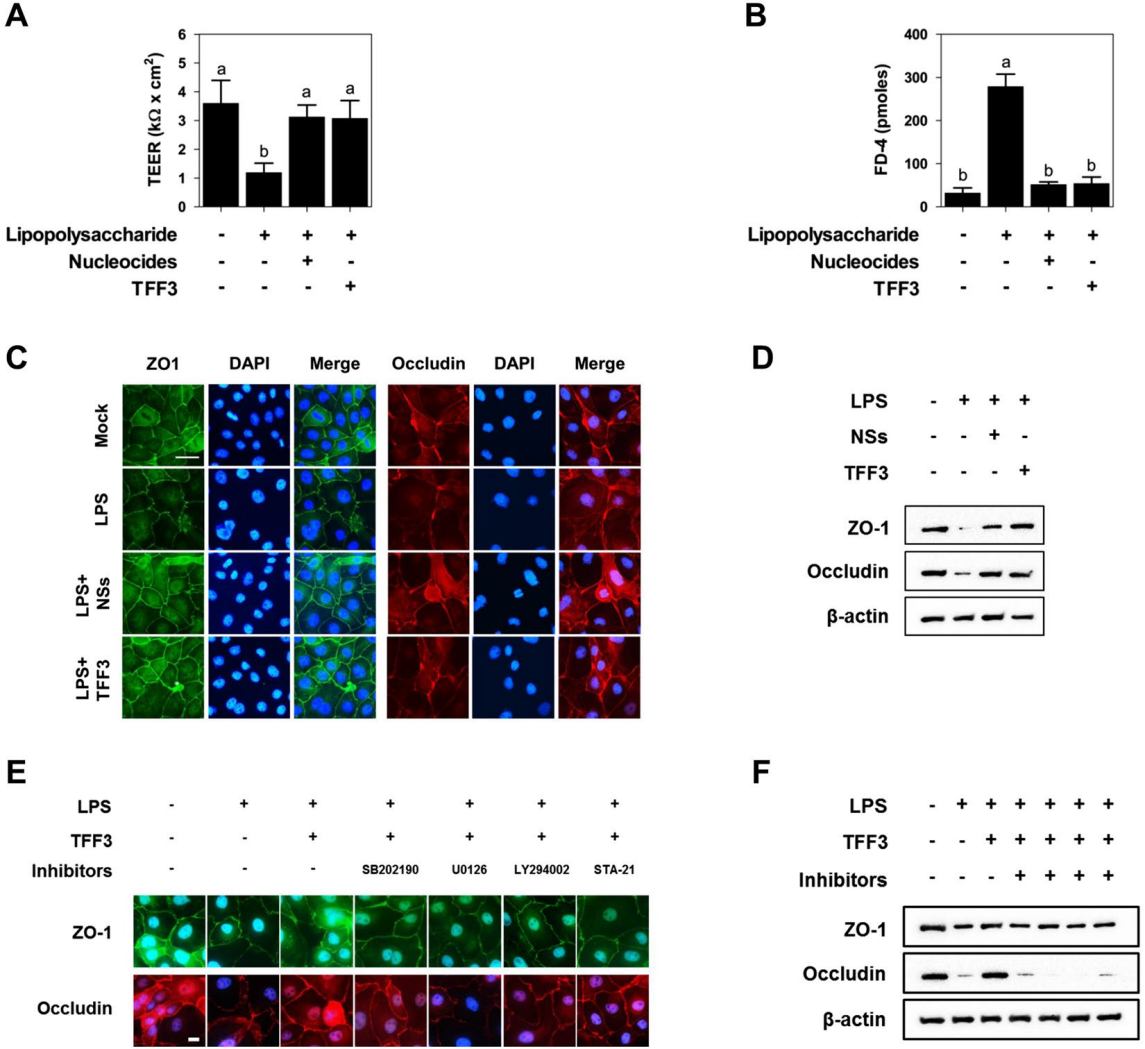
The protective effects of NSs and TFF3 on intestinal barrier function under LPS challenge. (**A**) Treatment with NSs and TFF3 increased transepithelial-electrical resistance (TEER) under LPS challenge in IPEC-J2 cells (n = 3). (**B**) Treatment with NSs and TFF3 increased the permeability of fluorescein isothiocyanate-labeled dextrans of 4 kDa (FD-4) under LPS challenge in IPEC-J2 cells (n = 3). Lowercase letters (a,b) indicate significant differences between treatments based on Duncan’s multiple range tests. Error bars indicate standard errors (SEs) of triplicate analyses. Immunofluorescence staining (**C**) and western blotting (**D**) showing the effects of NSs and TFF3 on the expression of tight junction proteins in LPS-challenged IPEC-J2 cells. Nuclei were stained with DAPI. Immunofluorescence staining (**E**) and western blotting (**F**) indicated that TFF3 improved intestinal barrier function via PI3K/Akt, ERK1/2, p38, and JAK/STAT signaling pathways. LY2944002, U0126, SB202190, and STA-12 were used as inhibitors of the respective signaling pathways. Error bars indicate standard errors (SEs) of triplicate analyses.

To investigate the effects of NSs and TFF3 treatment on the intestinal barrier function, we analyzed transepithelial electrical resistance (TEER), permeability, and tight junction protein expression in IPEC-J2 cells. IPEC-J2 cells treated with lipopolysaccharide (LPS) for 1 h showed reduced TEER compared with that in the untreated control, and NS- and TFF3-treated IPEC-J2 cells challenged with LPS showed increased TEER (Fig. 5A). The permeability of FD-4 in IPEC-J2 cells treated with LPS for 1 h was significantly increased compared with that in the control, and treatment with NS and TFF3 in LPS-challenged IPEC-J2 cells decreased the permeability of FD-4 in IPEC-J2 monolayers (Fig. 5B).

The results of immunocytochemistry and western blotting assay demonstrated that ZO-1 and occludin protein expression levels were significantly decreased in IPEC-J2 cells treated with LPS for 1 h compared with that in control cells and that treatment with NSs or TFF3 in LPS-challenged cells preserved the expression of ZO-1 and occludin (Fig. 5C and D). Next, we investigated whether TFF3 increased intestinal barrier function via PI3K/Akt, ERK1/2, p38, and JAK/STAT signaling pathways and promotion of tight junction protein expression. Importantly, TFF3 treatment preserved the expression of ZO-1 and occludin in LPS-treated IPEC-J2 cells; however, treatment with inhibitors of PI3K/Akt, ERK1/2, p38, and JAK/STAT blocked the role of TFF3 in modulation of ZO-1 and occludin expression in LPS-treated IPEC-J2 cells (Fig. 5E and F). These results suggested that NSs improved intestinal barrier function through induction of TFF3-mediated PI3K/Akt, ERK1/2, p38, and JAK/STAT signaling pathways in small intestinal epithelial cells.

### NT-mediated SAM pointed domain containing ETS transcription factor (SPDEF) induction regulated the expression of TFF3

**Figure 6:**
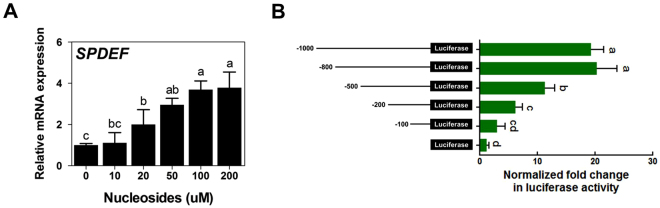
Nucleosides regulated the expression of *SPDEF*. (**A**) Effects of NSs on the expression of *SPDEF* in IPC-J2 cells. Significant differences between the control (0 μM) and treatment groups are indicated as *P < 0.05. Error bars indicate standard errors (SEs) of triplicate analyses. (**B**) Analysis of porcine TFF3 (pTFF3) promoter activity. pTFF3 promoters with different lengths (−1000, −800, −500, −200, and −100) were transfected into IPEC-J2 cells (n = 3). The relative luciferase activity was calculated as the ratio of firefly luciferase to Renilla luciferase. Lowercase letters (a,b,c and d) indicate significant differences between treatments based on Duncan’s multiple range tests. Error bars indicate standard errors (SEs) of triplicate analyses.

We then investigated the effects of NS treatment on activation of the transcription factor *SPDEF*, which directly regulates the expression of TFF3 in intestinal epithelial cells. Following 24 h of incubation of IPEC-J2 cells with varying concentrations of NSs (10, 20, 50, 100, and 200 µM), SPDEF expression levels were elevated in a concentration-dependent manner (Fig. 6A). Moreover, at concentrations of 100 and 200 µM, NSs markedly stimulated the expression of *SPDEF* in IPEC-J2 cells.

**Figure 7:**
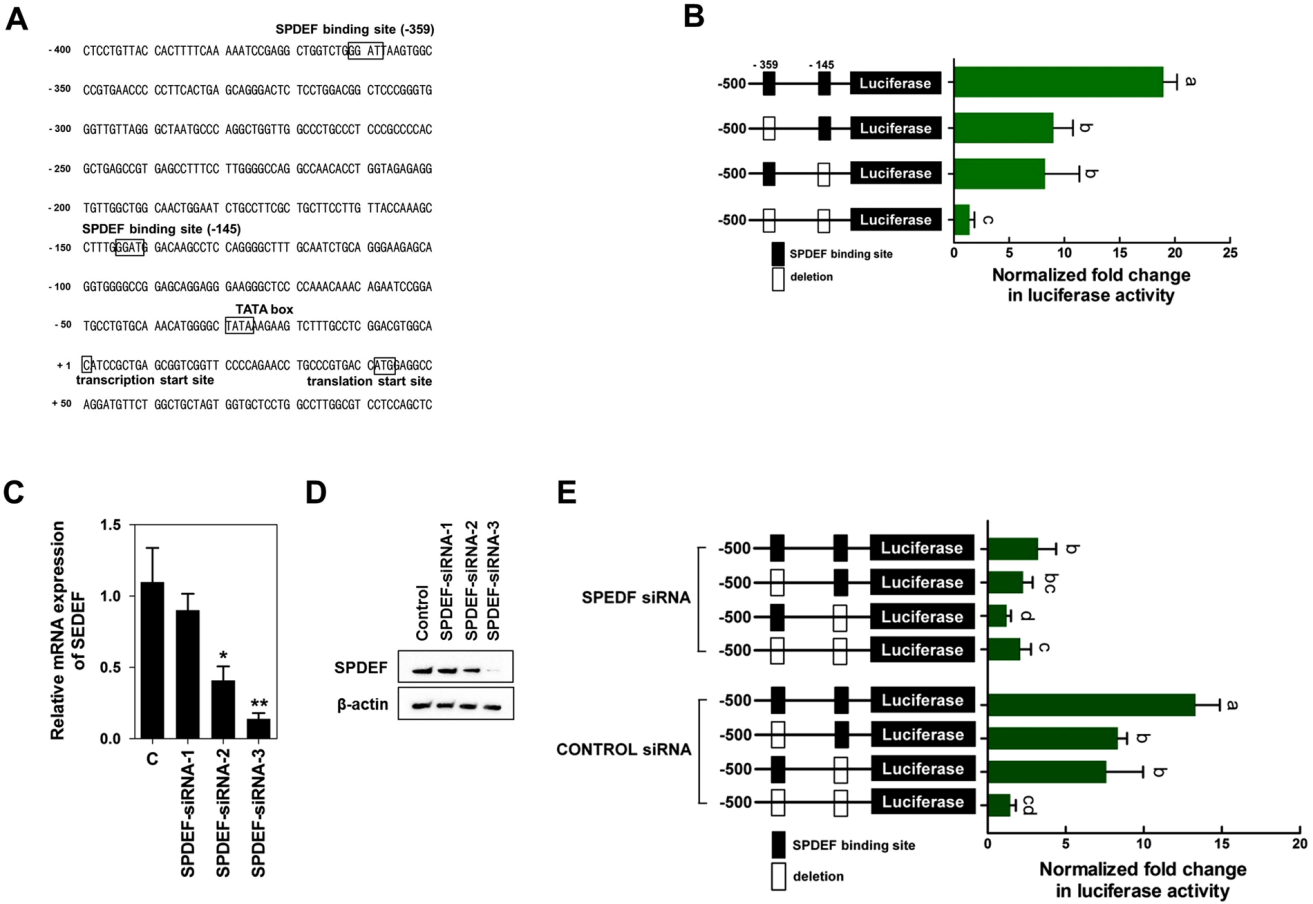
Nucleotide-mediated SPDEF induction regulated the expression of TFF3. (**A**) Nucleotide sequence of the core promoter region for the *pTFF3* gene. The numbering of the sequence is relative to the transcription start site. Putative binding sites for the transcriptional factors are boxed and labeled above. (**B**) Deletion analysis of the *pTFF3* promoter. Putative SPDEF binding sites (−359 and −145) were deleted and transfected into IPEC-J2 cells (n = 3). The relative luciferase activity was calculated as the ratio of firefly luciferase to Renilla luciferase. Lowercase letters (a,b and c) indicate significant differences between treatments based on Duncan’s multiple range tests. Error bars indicate standard errors (SEs) of triplicate analyses. qRT-PCR (**C**) and western blotting (**D**) indicated that SPDEF expression was suppressed in IPEC-J2 cells using siRNAs. siRNAs were introduced into IPEC-J2 cells by RNAiMAX. Nonspecific siRNA having no complementary sequence in the porcine genome was used as the control. Significant differences between the control and treatment groups are indicated as *P < 0.05 and **P < 0.01. Error bars indicate standard errors (SEs) of triplicate analyses. (**E**) The effects of SPDEF downregulation on transcription from the *pTFF3* promoter. Putative SPDEF binding sites (−359 and −145) were deleted with SPDEF siRNA-3 or control siRNA and transfected into IPEC-J2 cells (n = 3). The relative luciferase activity was calculated as the ratio of firefly luciferase to Renilla luciferase. Lowercase letters (a,b, and c) indicate significant differences between treatments based on Duncan’s multiple range tests. Error bars indicate standard errors (SEs) of triplicate analyses.

Next, we cloned DNA sequences with different lengths upstream of the porcine *TFF3* (*pTFF3*) gene into the firefly luciferase plasmid, and relative luciferase activity was detected using a dual luciferase reporter gene system (Fig. 6B). The relative luciferase activity was markedly stimulated in the presence of the upstream −1000, 800, and 500 sequence of the *pTFF3* gene compared with that of the control. Two regions (upstream of −359 and −145) of the SPDEF binding sequence (GGAT) were identified within the sequence at −500 upstream of the *pTFF3* gene (Fig. 7A). Single or dual deletion of the SPDEF binding sequence (upstream of −359 and −145) reduced relative luciferase activity compared with that of the control (Fig. 7B). From these results, we suggested that the region from 500 to 100 bp upstream was essential for the basal transcriptional activity of the *pTFF3* promoter.

We next examined whether the expression of TFF3 was altered by knockdown of SPDEF. Three siRNA sequences against porcine SPDEF siRNAs were designed, and we confirmed that these siRNAs knocked down SPDEF in IPEC-J2 cells compared with that in control cells transfected with nonspecific siRNA with no homology to porcine sequences (Fig. 7C and D). The knockdown efficiencies of siRNA-1, siRNA-2, and siRNA-3 against SPDEF were 19.74% ± 19.96%, 68.97% ± 16.99% (*P* < 0.05), and 95.95% ± 7.06% (*P* < 0.01), respectively. We used SPDEF-siRNA-3 for further experiments. Repression of *SPDEF* expression reduced the relative activity of the *pTFF3* promoter after single or dual deletion of the *SPDEF* binding sequence (upstream of −359 and −145) compared with that in the control (Fig. 7E). These results suggested that the transcription factor SPDEF directly regulated TFF3 expression via binding to the promoter region.

### Dietary NT supplementation improved the growth performance and villus height of the small intestine in weaned pigs

**Figure 8:**
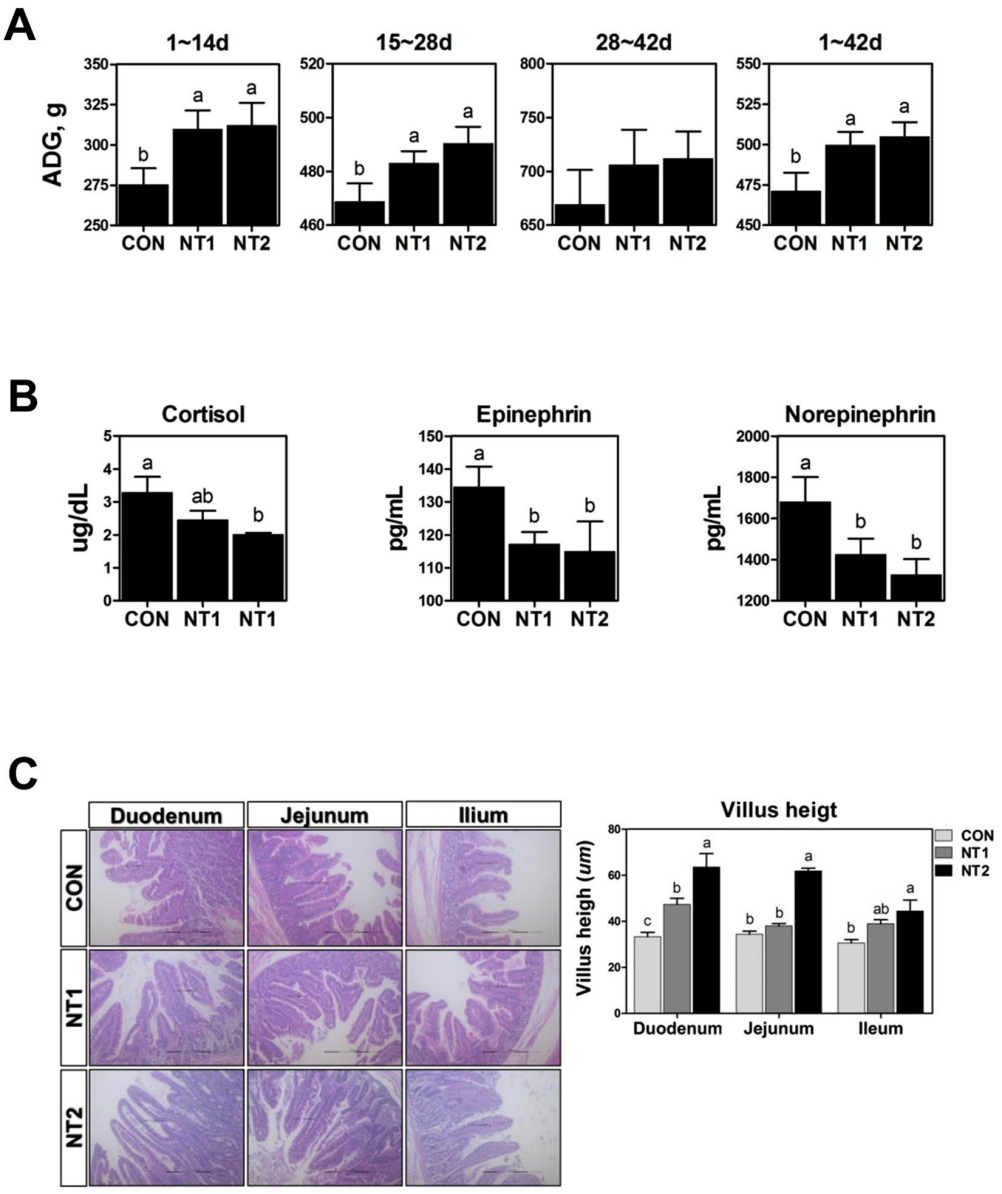
Dietary nucleotide supplementation improved growth performance and villus height of the small intestine in weaned pigs. Weaned pigs were randomly allocated into three groups: control (CON), a corn-soybean meal-based control; NT1, CON + 0.5% NTs; and NT2, CON + 1% NTs. (**A**) Average daily gain (ADG) of pigs in the three groups over time during the experiment. (**B**) At the end of the feeding trial (day 42), the levels of cortisol, epinephrine, and norepinephrine were determined by ELISA (n = 10). (**C**) On day 14, the duodenum jejunum, and ileum were sectioned and stained with H&E (n = 24). Lowercase letters (a, b, and c) indicate significant differences between treatments based on Duncan’s multiple range tests. Error bars indicate standard errors (SEs) of analyses.

To investigate the effects of NT supplementation on growth performance, serum stress levels, and development in the small intestine, we performed a feeding trial in weaned pigs. Pigs fed 0.05% and 0.1% NTs had higher average daily gain (ADG) than those fed the control diet during phases I (days 1–14), II (days 15–28), and IV (days 1–42; *P* < 0.05; Fig. 8A). At the end of the feeding trial (day 42), the level of cortisol was decreased in pigs fed 0.1% NTs, and the levels of epinephrine and norepinephrine were decreased in pigs fed 0.05% and 0.1% NTs (*P* < 0.05; Fig. 8B). Next, we investigated whether NT supplementation affected villus height in the duodenum, jejunum, and ileum on day 14. Pigs fed 0.1% NTs had higher villus heights in the duodenum, jejunum, and ileum compared with those in the control on day 14 (*P* < 0.05; Fig. 8C). From these results, NT supplementation improved growth performance, reduced stress, and increased villus height in the duodenum, jejunum, and ileum in weaned pigs *in vivo*.

## Discussion

In animal production, particularly in mammals, weaning is an important physiological and stressful event that can cause intestinal and immune system malfunction, resulting in reduced feed intake, growth retardation, and susceptibility to diseases, representing a major problem in livestock production^1,2^. When narrowing the focus to the small intestine, the weaning transition affects intestinal morphology, digestive and metabolic enzymes, inflammatory cytokines, and intestinal barrier function, resulting in intestinal villus atrophy, crypt hyperplasia, and increased intestinal permeability and promoting pathogen entry into the intestinal epithelium^11,12^. Although in-feed antibiotics are used for overcoming weaning-related problems, many candidates have been proposed as alternatives to antibiotics due to the need for reduced antibiotic use. Among others, NTs are one candidate that may have the potential to reduce weaning-related problems because dietary supplementation with NTs may exert beneficial effects on growth performance, small intestinal function, and immune responses^13,14^. However, the molecular mechanisms of NT-mediated intestinal development and function during the weaning transition have yet to be investigated in detail.

Thus, in the present study, we investigated the gene expression profiles in intestinal tissue with or without NT treatment and identified 748 annotated DEGs in the weaning transition. Among these DEGs, we selected TFF3, the intestinal trefoil factor; TFF3 is a peptide secreted from the normal mucous epithelium and is synthesized in mucin-producing cells, such as goblet cells, in the intestinal epithelium^15^. Consistent with previous findings, our results demonstrated that immunoreactive TFF3 protein was detected within goblet cells in the duodenum, jejunum, and ileum along the overlying mucosal surface. Additionally, we identified genes that were affected by dietary NT supplementation and further investigated the effects of exogenous NT treatment on TFF3-mediated functions in intestinal epithelial cells. Under physiological conditions, exogenous NTs have a limited capacity to pass through the intestinal epithelial cell membrane due to their high negatively charged phosphate group and the absence of a transport system for NTs^16^. Since only NSs can pass through the intestinal epithelial cell membrane, exogenous NTs need to be enzymatically hydrolyzed before absorption^8^. Thus, in the present study, we used NSs for further experiments *in vitro*. We demonstrated that treatment with various concentrations of NSs affected TFF3 induction in a concentration-dependent manner.

Intestinal wound healing is a repair process that is dependent on the precise balance of migration (restitution), proliferation, and differentiation in epithelial cells adjacent to the injured area, caused by toxic luminal substances, normal digestion, inflammation, interactions with microbes, oxidative stress, and pharmaceuticals^17^. In the context of mucosal damage, epithelial cells neighboring the wounded area rapidly migrate into the denuded area to restore barrier integrity, proliferate to increase the pool of enterocytes, and mature or differentiate to maintain the mucosal barrier function^17,18^. In the present study, we demonstrated that exogenous NSs and TFF3 treatment induced intestinal epithelial cell migration and that NSs but not TFF3 induced intestinal epithelial cell proliferation in a time-dependent manner. Additionally, NS and TFF3 treatment increased MMP2, MMP9, and RND3 expression and decreased CDH1 expression, demonstrating the effects of these treatments on wound healing-related genes. A number of reports have demonstrated that TFF3, which is secreted by goblet cells in the small and large intestines, has an important role in wound healing and repair of the intestinal mucosa, which is generally mediated by epithelial cell migration^19,20^. In previous studies, recombinant TFF3 administration was found to effectively moderate colitis in humans, and TFF3-deficient mice were found to be more susceptible to dextran sulfate sodium-induced colitis^21^. In rapidly dividing mucosal cells in the intestine, exogenous NT supplementation is important for intestinal development due to lack of *de novo* synthesis^22^. Consistent with the present study, dietary exogenous NT supplementation has been shown to increase intestinal cell proliferation in pigs and activate cell proliferation and differentiation in intestinal epithelial cells *in vitro*^13,23,24^. Thus, we suggest that dietary NTs may activate TFF3 under certain conditions to reduce *de novo* synthesis or stress, such as weaning, in order to enhance the wound healing process in the intestine.

In this study, we found that TFF3-mediated intestinal epithelial wound repair was regulated by various signaling pathways, such as PI3K/Akt, ERK1/2, p38, and JAK/STAT pathways, under NS-treated conditions in IPEC-J2 cells. In fact, various growth factors, cytokines, and signaling pathways are involved in intestinal epithelial wound repair, which can be promoted by different signaling pathways, such as transforming growth factor (TGF)-β-dependent and -independent pathways^17,25^. In previous studies, epidermal growth factor, fibroblast growth factor, and hepatocyte growth factor were found to promote intestinal epithelial wound repair through a TGF-β-dependent pathway^25^. In contrast, trefoil factors and galectins have been shown to promote intestinal epithelial wound repair through a TGF-β-independent mechanism^20,26^. Among these factors, TFF3 has been shown to promote the wound repair of gastric mucosal epithelial cells and preserve gastric mucosal epithelial integrity after damage is mediated by activation of the PI3K/Akt, ERK1/2, and p38 mitogen-activated protein kinase signaling pathway^27,28^. Thus, we verified that TFF3-mediated intestinal epithelial wound repair was enhanced by the JAK/STAT signaling pathway. From these results, we suggested that TFF3-mediated epithelial wound repair was promoted by NS treatment via the PI3K/Akt, ERK1/2, p38, and JAK/STAT signaling pathways.

The intestinal epithelium is lined by a single layer of cells that acts as a selective filter, allowing the translocation of essential dietary nutrients, electrolytes, and water from the intestinal lumen into the circulation. These cells also act as a barrier to prevent the passage of harmful intraluminal entities, including dietary antigens, microorganisms, and toxins^29^. The function of the intestinal barrier is mediated through the formation of a selectively permeable barrier across and between epithelial cells; this barrier is involved in the transport of water, ions, and macromolecules through transcellular and paracellular pathways^30^. The transcellular pathway is generally associated with the movement of nutrients, including amino acids, electrolytes, short chain fatty acids, and sugars, which are predominantly regulated by selective transporters through epithelial cells either by active or passive transport. In contrast, the paracellular pathway is associated with the transport of macromolecules in the space between epithelial cells and is regulated by intercellular complexes localized at the apical-lateral membrane junctions, including adherens junctions (AJs) and tight junctions (TJs)^30,31^. Maintenance of optimal intestinal barrier function is critical for animal health and growth performance because disruption of intestinal barrier function by the weaning process results in an increase in the intestinal permeability of pathogens, toxins, intestinal microbiota, and dietary antigens, followed by heightened bacterial translocation and inflammation and possibly the onset of various enteric and systemic disorders^32^. In the present study, we induced abnormal intestinal barrier function using LPS; this caused disruption of the apical-lateral membrane junctions between epithelial cells and allowed us to evaluate the effects of NSs and TFF3 on TEER, intestinal permeability, and tight junction protein expression. Following LPS treatment, NS and TFF3 treatment increased TEER and expression of tight junction proteins and decreased membrane permeability via PI3K/Akt, ERK1/2, p38, and JAK/STAT signaling pathways. Thus, our findings suggested that NSs improved intestinal barrier function through induction of TFF3-mediated PI3K/Akt, ERK1/2, p38, and JAK/STAT signaling pathways in small intestinal epithelial cells.

Under rapid growth conditions, such as the neonatal period and tissue repair after injury, during which *de novo* NT synthesis occurs, NTs can be considered semi-essential nutrients^33,34^. Additionally, exogenous NSs affect the expression and activity of several transcription factors involved in cell growth, differentiation, apoptosis, immune response, and inflammation in intestinal epithelial cells, indicating that exogenous NTs have been shown to modulate many genes^35^. In the present study, we focused on SPDEF as an NT-mediated DEG and tested whether SPDEF regulated the expression of TFF3 in intestinal epithelial cells. We confirmed that NS treatment elevated the expression of SPDEF in a concentration-dependent manner and that SPDEF directly regulated the expression of TFF3 through binding to the upstream region of the *TFF3* gene. The expression of TFF3 is regulated by many different transcription factors, including specificity protein 1, caudal-related homeobox transcription factor 2, and nuclear factor kappa-light-chain-enhancer of activated B cells^36,37^. The present study demonstrated that SPDEF, which belongs to the ETS family of transcription factors, regulated the expression of TFF3 in intestinal epithelial cells. Notably, SPDEF is a transcriptional coregulator of atonal BHLH transcription factor 1, a critical intestinal secretory lineage-specific transcription factor controlling cellular differentiation and maturation of intestinal epithelial cells; through this pathway, SPDEF inhibits the proliferation of intestinal progenitors and promotes terminal differentiation into intestinal goblet cells^38,39^. Collectedly, our findings suggested that NS treatment induced the expression of SPDEF to directly regulate TFF3 expression via binding to the promoter region in the context of rapid wound repair-related stress caused by the weaning transition in intestinal epithelial cells.

The maintenance of adequate piglet performance during the weaning process is a primary economic target in pig production because the weaning process is associated with decreased digestive function, neuroendocrine integrity, and immune status. In particular, the shift from milk to dry feed during weaning induces low feed intake, resulting in growth retardation and alterations in gut architecture^2,40^. The present study revealed the effects of NT treatment on intestinal epithelial cells; NTs induced the expression of SPDEF, resulting in modulation of TFF3-mediated wound healing and intestinal barrier function via the PI3K/Akt, ERK1/2, p38, and JAK/STAT signaling pathways. To confirm the effects of NT treatment on intestinal epithelial cells *in vitro*, we performed a feeding trial and examined growth performance, serum stress levels, and development of the small intestine in weaned pigs. We found that NT supplementation improved growth performance, serum stress levels, and intestinal morphology. Consistent with these findings, dietary NT supplementation at greater than physiological quantities enhanced the adaptive capabilities of piglets to weaning stress and improved growth performance and plasma cortisol levels during the first 2 weeks after weaning^14,40^. Thus, the findings of the present study suggested that NTs could be used as a feed additive in piglets during the weaning process.

**Figure 9:**
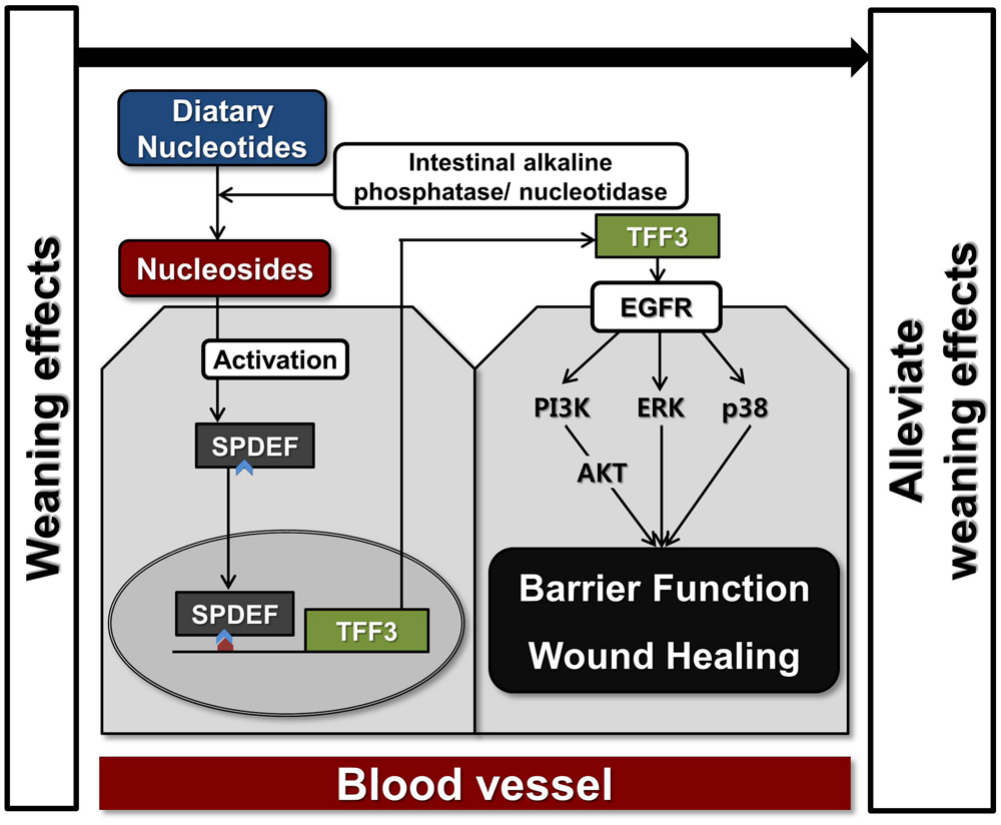
Schematic illustrating the current working hypothesis regarding the regulation of SPDEF-mediated TFF3 by NT treatment on intestinal development during the weaning process. NTs induced the expression of SPDEF, which regulated TFF3 expression, resulting in modulation of TFF3-mediated wound healing and intestinal barrier function via the PI3K/Akt, ERK1/2, p38, and JAK/STAT signaling pathways.

In conclusion, we identified 748 DEGs using a nutrigenomics analysis of NT treatment during intestinal development in piglet undergoing weaning. We also revealed that NTs induced the expression of SPDEF, which regulated TFF3 expression, resulting in modulation of TFF3-mediated wound healing and intestinal barrier function via the PI3K/Akt, ERK1/2, p38, and JAK/STAT signaling pathways (Fig. 9). In a feeding trial, NT supplementation improved growth performance, serum stress levels, and intestinal morphology. Collectively, these data indicated that NTs could be used as a feed additive in piglets. This study may help improve our understanding of the molecular mechanism of intestinal development during the weaning process in pigs.

## Methods

All experiments were approved by the Animal Care Committee of Dankook University and were conducted in accordance with the guidelines for the care and use of experimental animals for research at Dankook University.

### Experimental animals and diet

A total of 120 crossbred weaned pigs ([Yorkshire × Landrace] × Duroc) with an average body weight of 8.32 ± 1.34 kg were used in a 6-week trial. The pigs were sorted into pens with five pigs per pen and eight pens per treatment. The treatments were as follows: control, a corn-soybean meal-based control; NT1, control plus 0.5% NTs; and NT2, control plus 1% NTs. All diets were formulated to meet or exceed the NRC (2012) nutrition requirements (Supplemental Table 5). All pigs were housed in an environmentally controlled room with a slatted plastic floor. Each pen was equipped with a self-feeder and nipple waterer to allow ad libitum access to feed and water throughout the experimental period. Temperature during week 1 was maintained at 32 °C and was lowered 2.5 °C each week thereafter. Individual pig body weights were recorded at the beginning of the experiment and then on days 14, 28, and 42, and feed consumption was recorded for each pen during the experiment to calculate ADG. Blood samples (10 mL) were collected via anterior vena cava puncture from 10 random pigs per treatment on day 42. Serum was separated by centrifugation at 4,000 × *g* for 30 min at 4 °C, and aliquots were stored at −4 °C for subsequent determination of cortisol, epinephrine, and norepinephrine levels. Serum cortisol concentrations were determined using a standardized solid phase radioimmunoassay kit (Dianostic Products Corporation, Los Angeles, CA, USA). Serum epinephrine and norepinephrine levels were quantified using an ion-exchange purification procedure followed by high-performance liquid chromatography with electrochemical detection.

### Reagents

Dulbecco’s modified Eagle’s medium (DMEM), Opti-MEM, phosphate-buffered saline (PBS), fetal bovine serum, insulin-transferrin-selenium-X, and penicillin-streptomycin mixture were purchased from Life Technologies (Carlsbad, CA, USA). NSs (cytidine, adenosine, guanosine, and thymidine), LPS, and fluorescein isothiocyanate-labeled dextran (molecular mass of 4 kDa, FD4) were purchased from Sigma-Aldrich (St. Louis, MO, USA). Recombinant TFF3 was purchased from Cloud-Clone Corp. (Katy, TX, USA). Lipofectamine 2000 and RNAiMAX reagent were purchased from Invitrogen (Carlsbad, CA, USA). The pGL3-Basic (Promega, Madison, WI, USA) and pcDNA3.1 (+) (Thermo Fisher Scientific, Waltham, MA, USA) plasmids were used as cloning vectors. Primary antibodies specific for TFF3 (Antibodies Online), β-actin (Abcam, Cambridge, UK), ZO-1 (Thermo Scientific), occludin (MybioSouce), phospho-AKT (Cell Signaling Technology), AKT (Cell Signaling Technology), phospho-p38 (Cell Signaling Technology), p38 (Cell Signaling Technology), phospho-STAT3 (Cell Signaling Technology), STAT3 (Cell Signaling Technology), phospho-ERK1/2 (Cell Signaling Technology), and ERK1/2 (Cell Signaling Technology) were used for western blotting, immunohistochemistry, and immunofluorescence. The secondary antibodies were purchased from Abcam.

### Expression profiling analysis

During the feeding trial, three piglets per treatment (control and NT2) were sedated with xylazine and ketamine and euthanized with an overdose of pentobarbital administered via an ear vein on day 14. The abdominal cavity of the piglets was opened, and intestinal samples were removed from the small intestine. All intestinal samples were pooled, frozen in liquid nitrogen, and stored at –80 °C. Total RNA was isolated using TRIzol reagent (Invitrogen). RNA quality was assessed using an Agilent 2100 bioanalyzer with an RNA 6000 Nano Chip (Agilent Technologies, Amstelveen, The Netherlands), and RNA quantification was performed using an ND-2000 Spectrophotometer (Thermo Inc., DE, USA). For library preparation and sequencing, library construction was performed using a SENSE 3′ mRNA-Seq Library Prep Kit (Lexogen, Inc., Austria) according to the manufacturer’s instructions. In brief, each 500 ng total RNA was prepared, and an oligo-dT primer containing an Illumina-compatible sequence at its 5′ end was hybridized to the RNA; reverse transcription was then performed. After degradation of the RNA template, second strand synthesis was initiated by a random primer containing an Illumina-compatible linker sequence at its 5′ end. The double-stranded library was purified using magnetic beads to remove all reaction components. The library was amplified to add the complete adapter sequences required for cluster generation. The finished library was purified from PCR components. High-throughput sequencing was performed by single-end 75 sequencing using a NextSeq. 500 instrument (Illumina, Inc., USA).

### Cell culture and treatments

The nontransformed porcine intestinal epithelial cell line IPEC-J2 (DSMZ, Braunschweig, Germany), originally isolated from the jejunal epithelium of a piglet, was cultured in DMEM (high glucose) supplemented with 5% fetal bovine serum, 1% insulin-transferrin-selenium-X, and 1% (v/v) penicillin-streptomycin mixture^41^. Cells were maintained at 37 °C in a humidified atmosphere of 5% CO_2_. IPEC-J2 cells were incubated with various concentrations of NSs, TFF3, or expression plasmids for different intervals as indicated in the figure legends.

### qRT-PCR and western blotting

For qRT-PCR, total RNA (1 µg) was used for complementary DNA synthesis with a Maxima First-strand cDNA Synthesis Kit (Life Technologies). The primers for qRT-PCR for each gene transcript were designed using Primer3 (http://frodo.wi.mit.edu/; Supplemental Table 6). qRT-PCR was performed using a 7500 Fast Real-time PCR System (Applied Biosystems) with the following conditions: 94 °C for 3 min, followed by 40 cycles at 94 °C for 30 s, 59–61 °C for 30 s, and 72 °C for 30 s. Melting curve profiles were analyzed for the amplicons. qRT-PCR data were normalized to the expression of glyceraldehyde 3-phosphate dehydrogenase (*GAPDH*), an endogenous control gene, and calculated using the 2^−ΔΔCt^ method, where ΔΔCt (cycle threshold) = ΔCt (treated) − ΔCt (control) and ΔCt = Ct of the target gene − Ct of *GAPDH* (treated or control, respectively)^42^.

For western blotting, IPEC-J2 cells were immediately washed with ice-cold PBS two times, and the cell pellet was lysed with Lysis Buffer (Cell Signaling Technology) containing Protease Inhibitor Cocktail (Roche, Basel, Switzerland). Cellular debris was removed by centrifugation at 14,000 × *g* at 4 °C for 10 min. Supernatants were collected, and the protein concentration was measured using a Pierce BCA Protein Assay Kit (Thermo Fisher Scientific). Protein from each sample (20 μg) was electrophoresed by sodium dodecyl sulfate polyacrylamide gel electrophoresis on 10% gels for 1 h at 120 V and transferred to nitrocellulose membranes (Millipore, Billerica, MA, USA) using a Mini-PROTEAN Tetra Cell (Bio-Rad, Hercules, CA, USA). After blocking for 1 h, the membrane was incubated with appropriate primary antibodies overnight at 4 °C. After being washed with TBST three times, the membrane was incubated in appropriate secondary antibodies for 1 h at room temperature. The immunocomplexes were detected using an ECL Select Western blotting detection reagent (GE Healthcare, Little Chalfont, UK) and were imaged using a ChemiDoc Imaging System (Bio-Rad). Densitometric quantification was performed using Image J software (National Institutes of Health, Bethesda, MD, USA).

### Immunohistochemistry and immunofluorescence

During the feeding trial, small intestine tissues were collected on day 14. The duodenum, jejunum, and ileum were fixed overnight in 4% formaldehyde, embedded in paraffin, sectioned at 5 μm, and stained with hematoxylin-eosin (H&E). For measurement of villus height, four villi were randomly selected from each cross-section. From a total of 24 villus heights from six cross-sections, the villus height from the tip to base, excluding the intestinal crypt, was measured. For immunohistochemistry, sections were deparaffinized using xylene and rehydrated in a series of ethanol concentrations. Endogenous peroxidases were blocked using methanol with 3% H_2_O_2_. For antigen retrieval, tissues were boiled in citrate buffer (pH 6.0) using a microwave for 10 min. Subsequently, slides were incubated with primary antibody in PBS with 1% bovine serum albumin and 0.1% Triton X-100. Sections were then washed and incubated with a SignalStain Boost IHC Detection Reagent (Cell Signaling Technology) for 1 h. Slides were washed in PBS, and a SignalStain DAB Substrate Kit was used according to the manufacturer’s instructions.

For immunofluorescence, IPEC-J2 cell monolayers grown on glass coverslips were fixed in 4% paraformaldehyde. Following blocking with 2% bovine serum albumin in PBS, the cells were incubated with primary antibodies used at a dilution of 1:100 (ZO-1 and occludin) overnight. After repeated washing, fluorophore-conjugated secondary antibodies (Alexa Fluor 488 and Alexa Fluor 594) were applied for 1 h at room temperature in the dark, and the cells were washed again. Mounting was performed using VECTASHIELD Antifade Mounting Medium with DAPI (Vector Laboratories, Burlingame, CA, USA), and images were collected using a fluorescence microscope. Fluorescence intensities were quantified using the threshold function of Image J software.

### Cell proliferation and migration assays

IPEC-J2 cells were seeded in 96-well plates at a density of 1 × 10^4^ cells/well. The cell proliferation reagent WST-1 (Roche Applied Science, Indianapolis, IN, USA) was added to each well according to the manufacturer’s instructions. The absorbance of the dye after incubation was measured at a wavelength of 450 nm with background subtraction at 690 nm with a BioTek Synergy HTTR microplate reader (BioTek Instruments, Winooski, VT, USA). IPEC-J2 cells were cultured with or without NSs and TFF3 in 60-mm culture dishes until reaching confluence. Subsequently, straight scratches were made with a p200 pipette tip across the 60-mm culture dish, simulating a wound. Photographs were acquired at different intervals, as indicated in the figure legends.

### TEER and intestinal permeability

Confluent monolayers of IPEC-J2 cells were grown in 24-well Corning Transwell chambers (polycarbonate membrane, filter pore size 0.4 μm, area 0.33 cm^2^; Costar) and then treated with or without NSs and TFF3 for 24 h. The cells were washed twice and incubated with LPS (1 μg/μL) for 1 h. TEER was measured using an epithelial voltohm meter (World Precision Instruments, Sarasota, FL, USA). TEER values were calculated by subtracting the blank filter (90 Ω) and by multiplying the surface area of the filter. All measurements were performed on a minimum of triplicate wells.

When the IPEC-J2 monolayer was confluent (≥1 kΩcm²), the cells were treated with or without NSs and TFF3 for 24 h. The cells were washed twice and incubated with LPS for 1 h. The cells were then washed twice again. The permeability assay started when 500 μL of culture medium containing 1 mg/mL of FD-4 (Sigma-Aldrich) was added to the apical chamber. The basolateral chamber was filled with 1.5 mL culture medium (37 °C, 5% CO_2_). FD-4 was allowed to permeate overnight (18 h) from the apical to the basolateral chamber. Subsequently, 100 μL of the basolateral chamber medium was transferred to a 96-well plate to measure the amount of permeated FD-4 using a flour-spectrophotometer (Ex/Em: 490/520 nm).

### Vector construction, knockdown, and luciferase assay

For analysis of mRNA functionality, we designed siRNAs against SPDEF (Supplemental Table 7) and performed transfection in IPEC-J2 cells using RNAiMAX. Briefly, 7.5 μL RNAiMAX reagent diluted in 100 μL Opti-MEM solution was incubated for 5 min at room temperature, and the mixture was added to 75 nM SPDEF siRNA diluted in 100 μL Opti-MEM solution. The mixture of RNAiMAX and siRNAs was then added drop-wise to IPEC-J2 cells in culture medium lacking antibiotics. Total RNA was extracted and analyzed by qRT-PCR 48 h after transfection. The different lengths of the upstream sequences of the *TFF3* gene and −500 upstream sequence containing deletions of the SPDEF binding site were synthesized by a commercial company (Bioneer Ltd.). Each upstream region of pTFF3 was subcloned into the multiple cloning region of the pGL3-Basic Vector. The ORF sequence was synthesized and cloned into the pcDNA3.1 (+) vector. Transfection was performed according to the manufacturer’s protocol.

For luciferase assays, the cells were harvested 48 h after transfection, washed with cold PBS, and used in a Luciferase assay system (Promega), according to the manufacturer’s instructions. Briefly, cells were resuspended with 100 μL of diluted Passive Lysis Buffer, the extracts were centrifuged at 13,000 × *g* for 5 min, and the supernatants were collected. The activities of firefly and Renilla luciferases were measured using a GLOMAX 20/20 Luminometer (Promega). All measurements were normalized to Renilla activity.

### Statistical analysis

Data were analyzed with the general linear model (PROC-GLM) procedure of SAS to determine the significance of differences between the treatments. Results are presented as means and standard errors (n ≥ 3, where n refers to the number of replicate experiments). A *p*-value of less than 0.05 indicated statistical significance. Significant differences between control and treatment groups are indicated as ***P < 0.001, **P < 0.01, and *P < 0.05. Significant differences between treatments were assessed by Duncan multiple range tests.

## Electronic supplementary material

Supplemental table 1

Supplemental table 2

Supplemental table 3

Supplemental table 4

Supplemental table 5

Supplemental table 6

Supplemental table 7

whole image of blots
