# Supplementary material for: Nucleotide-mediated SPDEF modulates TFF3-mediated wound healing and intestinal barrier function during the weaning process
Source: Sci Rep. 2018 Mar 19;8:4827. doi: 10.1038/s41598-018-23218-4 (PMC5859294; doi:10.1038/s41598-018-23218-4)

Nucleotide-mediated SPDEF modulates TFF3-mediated wound healing and intestinal barrier function during the weaning process

Sang In Lee and In Ho Kim*

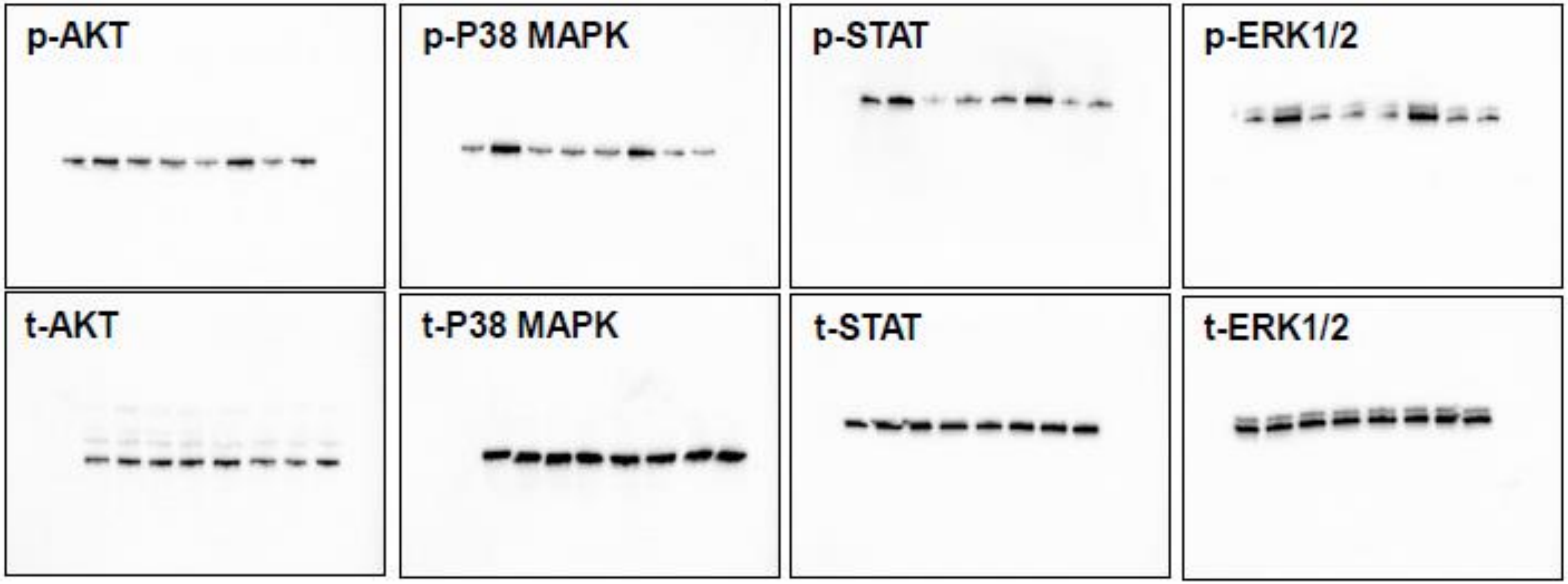

Supplement: Supplementary file 8 — whole image of blots [file 41598_2018_23218_MOESM8_ESM.pdf]
